# Cyclopædia of Practical Medicine

**Published:** 1836

**Authors:** 


### Art. VIII.—Cyclopædia of Practical Medicine.

*The Cyclopædia of Practical Medicine, comprising Treatises on the Nature and Treatment of Diseases, Materia Medica and Therapeutics, Medical Jurisprudence, etc., etc.* Edited by John Forbes, M. D., Alexander Tweedie, M. D., and John Conolly, M. D., 4 volumes, 8 mo. London, 1833–5.

Without having had occasion to consult this work extensively, we have perused a sufficient number of articles, to pronounce it a valuable addition to our libraries of theoretical and practical medicine. Its authors are more than 60 in number, and comprehend many of the most distinguished physicians of England, Ireland and Scotland—among whom we note, the able and eminent writers, whose names are put forth as the immediate editors of the work, and the no less reputable names of Bostock, Caswell, Cheyne, Clark, Elliotson, Hall, Hastings, Paris, Prichard, Stokes, the Thompsons, Williams, and many others. A work emanating from such men, may fairly be regarded as an expression of the medical mind of Great Britain and Ireland; and we are happy to find that expression, much more in consonance with the existing improved state of pathology and pathological anatomy, than it was, even but a few years since, in the same countries. A greater regard and affection for the remains of the dead, and a deeper feeling of solemnity on the subject of death, in England than France, formed, as we suppose, on a more serious and diffusive belief in the immortality of the soul among the people of the former than the latter kingdom, seems to have been a great cause of the earlier progress of pathological anatomy on the continent than the island, or in these United States, where Saxon principles and prejudices have been cherished on the same points, if not to the same degree, as in our mother country. We are happy to find, however, in the work before us, an evidence that the association of ideas, which led our ancestors into fastidiousness in regard to the body which is the tenement of an immortal soul, has been so modified, as that the medical men of Great Britian, are beginning to obtain much more frequent opportunities of inspecting the ravages of disease, and of reasoning from them to the morbid actions of which they are the vestiges, than in former times; and that the work before us, contains a great deal of sound pathology, based of course upon a comparison of morbid with healthy structures. We are decided admirers of the French school of pathology; but, with all the objurgation, which has, in modern times, been poured out, and often justly, upon British and American polypharmacy, we prefer it to the simple therapeutics of France. It was only necessary, to correct, modify and enlighten, the complex and energetic, *methodus medendi* of the former, by the pathological discoveries of the latter, to render it eminently efficient, and this has lately been to a great extent accomplished. To say nothing of other works, which might be cited, not a few of the treatises in that now under our notice, present a union of Gallic pathology, with Anglo-Saxon therapeutics, which we regard as highly auspicious of the future. As an example we shall transcribe a part of the article "PERITONITIS," by Dr. McAdam of Dublin, which, moreover, from its matter cannot fail to interest our readers.

"*Morbid Anatomy of Peritonitis.*—The alterations which the peritoneum presents after death from this disease are essentially the same as are found in cases of inflammation of other serous membranes. The morbid effects are greater or less, according to the intensity and duration of the disease. They are sometimes confined exclusively to the peritoneum, evidencing that this membrane may be partially or generally inflamed, without the subjacent tissues being affected; however, in some of the complicated forms of this disease, morbid lesions of the other intestinal tunics will be occasionally discovered to co-exist with those characteristic of peritonitis.

The following are the effects of inflammation of the peritoneum, as far as they are revealed by dissection:—1. increased vascularity and thickening of the peritoneum; 2. effusion of coagulable lymph, either in the form of flocculi, membranes, bands, or masses; 3. effusion, into the peritoneal cavity, of various fluids, serum, pus, or blood, mixed or separate; 4. gangrene; 5. tuberculous formations; 6. granulations on the peritoneal surface; 7. ulceration.

1. *Morbid Appearances in Acute Peritonitis.*—The first effects of a low degree of inflammatory action upon serous membranes appear to be simply an increased deposition of the serous fluid; and in this manner it is probable that a certain state of these membranes, which, if not actually inflammatory, closely borders upon it, is sometimes relieved; the increased quantity of fluid being afterwards absorbed, and the parts recovering their healthy relations.*

When inflammation is fully established, its earliest effect is increasd vascularity, which produces at first a slight degree of opacity of the membrane, and red points begin to appear on its surface, which may either occupy a small portion, or cover nearly the whole extent of the peritoneum; the surface of which at this time appears dry and shining, but on touching it an unctuous coating will be detected. Sometimes, instead of the red points, bloodvessels are developed, forming red striæ more or less numerous. As the inflammation advances, the small points become multiplied, coalesce, and from patches of variable extent, and the bloodvessels become more evident and numerous. In a more advanced stage, the redness is rendered more intense, and occupies a larger portion of the membrane; sometimes forming broad surfaces of inflammation, which run like bands along the course of the intestines, and are bounded by the adhesion which different portions of the bowels contract with each other. This redness is frequently arborescent, sometimes intermixed like network. The vascular injection has been supposed by some to exist in the arterial capillaries; but Dr. Armstrong observes, that whatever may be the case during life, it is after death chiefly seated in the venous capillaries; for on a minute inspection the small ramifications of the arteries may be seen empty, traversing the intermediate portion of intestine, like so many transparent lines. The degree of redness is ultimately influenced by the quantity of secretion being greater in those cases where there is least serum and lymph.†

Along with this redness we observe more or less thickening and opacity of the peritoneum; an effect produced not only by the hyperæmia of the inflamed part, but also by the effusion of serum, lymph, or both, into the subserous cellular tissue, which causes some degree of pulpiness, and a facility in separating the serous coat from the subjacent parts. The serous membrane itself is also thickened by the effused fluid penetrating between its laminæ, and separating them more or less from each other, and in some instances a slight

---

* *Abercrombie*, p. 3.

† M. Scoutetten asserts that this redness, when intense, is not owing to the distention of the bloodvessels, but to a sanguine exudation which is formed on the surface of the peritoneum, and which adheres strongly to it; and that the surface is uniformly red, and appears villous. Both causes probably concur to produce the effect.

Bichat, M. Gac, and others, have asserted that the absence of redness on the peritoneal surface after death may occur in cases where the membrane was inflamed during life. M. Scoutetten, (Archieves Generales, tom. iii. p. 501,) however, from some experiments he has performed on living animals, has come to a contrary conclusion; he asserts that the disappearance of redness from an inflamed *external* surface after death is owing to the pressure of the atmosphere, which has but a very modified influence on an *internal* tissue: and that, consequently, the characters of inflammation are very nearly the same in those tissues during life and after death; his experiments are highly ingenious, and would seem to justify the conclusion he has deduced from them, viz. *that an inflammation of an internal membrane will in every case leave increased redness after death.*

degree of emphysema exists, from the disengagement of air in the connecting cellular tissue; but when the inflammation is slight, this thickening is not apparent.

The intestines are much thicker and more massy, as well as the mesentry and the mescolon; and the omentum sometimes is rendered as thick as a person's hand; effects which arise from the extravasation of coagulable lymph into the cellular substance between the laminæ of peritoneum which forms them.*

Redness and thickening may be considered as the first effect of peritoneal inflammation; but it is accompanied or quickly followed by effusion of serum and lymph, which have been supposed to be separated simultaneously. Dr. Armstrong, however, seems to think that the lymph is first effused: but it would appear that the degree of intensity of the inflammation determines the nature of the effusion. When the inflammation is not very violent, serum seems to be the earliest product of the vessels of the affected part; but if the inflammatory action is very acute, lymph is often thrown out in the first instance. This effusion of lymph may take place a very short time after the commencement of inflammation; it is at first soft and gelatinous, afterwards becomes more consistent, and finally assumes the texture of a membrane of considerable tenacity. It generally soon becomes organized: Andral † observes that in some cases, twenty hours after the commencement of peritonitis, vessels can be traced and injected in this fibrous concretion, which has become a living texture; in other cases, after several months, no trace of organization can be found in these membranous layers. This coagulable lymph may assume various forms; it may either be deposited in a lamina of variable thickness, lining the peritoneal surfaces, agglutinating the intestines to each other, to the abdominal parietes, or to the omentum; or narrow bands of membrane, of variable length, may connect two or more coils of intestine together; or a mass of lymph of a prismatic form may lie between the intestinal convolutions, filling up their interstices, the anterior flat surface of which mass corresponds to the parietes of the abdomen, and the two other surfaces, slightly concave, are applied on the two contiguous intestines, and terminate in their interval by a rounded angle. The lymph may also be in the form of flocculi, or small shreds of membrane, floating in a serous fluid, or deposited on the free surface of the peritoneum when little or no fluid is found.

These false membranes are either of a white color, grey, or sometimes a little reddish; they vary in their thickness from a quarter to two or three lines. When the inflammation has lasted twenty or thirty days, these adhesions acquire a considerable degree of firmness; they are often productive of no inconvenience, but if very numerous, and uniting the coils of intestine very closely together, they may give rise to a modification of the form of the belly, an habitual tension in the abdomen, and more or less disturbance in the digestive functions. Dr. Abercrombie relates some cases where fatal ileus seemed to be induced by the operation of such causes, which appeared

---

* Baillie's Morbid Anatomy.

† Clinique Medicale.

to act by deranging the muscular power of the intestinal canal, or by inducing a strangulation of a portion of intestine.

Serous effusion, as we have before observed, may take place very early in this disease:* bnt in general it is not considerable in quantity until the affection has existed for some time. This, however, is not always the case; in some instances, after thirty-six or forty hours, there is a large quantity of fluid effused; it is generally accumulated in the inferior parts, unless retained in distinct cavities by adhesions. Its color varies very much; it is sometimes limped; and that is principally the case when the peritonitis has been partial;† at other times it is whitish, greyish, milky, or yellowish, and occasionally has all the sensible properties of pus. Dr. Abercrombie observes that the opaque milky deposition is commonly connected with alteration of the structure of the membrane, which in such instances presents a soft thickened appearance, resembling a part that had been boiled. The more common appearance of the peritoneum consists of a deposition of false membrane, co-existing either with the milky flocculent fluid, or with pus, or a fluid entirely limped. In the latter case, the deposition on the surface of the membrane will prevent the re-absorption of the fluid; so that the accumulation which might otherwise have disappeared will thus become a permanent cause of ascites, provided the disease does not prove speedily fatal.‡

This effusion is sometimes reddish, evidently from a mixture of the coloring matter of the blood; and in the hemorrhagic variety of peritonitis, large quantities of blood in a coagulated state have been found by M. Broussais in the peritoneal cavity, without any rupture of vessels being detected, accompanied with extensive ecchymoses of the cellular tissue which unites the peritoneum to the contiguous parts.

The quantity of fluid effused in peritonitis varies from a few ounces to several pounds; its consistence is sometimes that of water, without containing any albuminous clots: at other times it equals that of milk or the pus of the cellular tissue, and often contains the flocculi before mentioned.

The peritoneum is seldom found in a state of gangrene in its whole extent; but eschars of variable size, and of a greyish slate-color or blueish, are formed, which are easily lacerated, and exhale a fetid odor. The surface of these eschars is often covered, with a greyish soft matter, little adherent, which appears to be the commencement of decomposition; these eschars sometimes pervade the entire thickness of the intestinal tunics, or the great epiploon, or extend deeply into the abdominal parietes.

---

* In some cases of peritoneal inflammation the secretion of this membrane is suspended, and it appears dry.

† Bichat observes that, when serous effusions are the consequence of an affection of a viscus, the serosity is limpid, transparent, and probably of the same nature with that which is exhaled in its natural state; but that, on the contrary, when the effusion depends on inflammation of serous membranes, the fluid is almost always altered. (*Roche et Sanson* Elements de Pathologie, tom. i. p. 552.)

‡ *Abercrombie*, p. 3.

There are certain brownish, black, or violet degenerations of the peritoneum which have been mistaken for gangrene, but are only the results of chronic irritation; they are easily distinguished by not being readily torn, by being destitute of the gangrenous odor, and by the great extent to which the peritoneum is discolored, the entire membrane being sometimes affected. These appearances are a common consequence of chronic peritonitis.

Ulceration very rarely occurs as a consequence of peritonitis; though not unfrequently the peritoneum is perforated in cases where the ulcerative process, commencing in the mucus membrane, erodes the other coats of the intestines; this naturally leads us to consider peritoneal ulcers as of two k nds:—1. *primitive ulcers*, or those which occur primarily in the peritoneum as a direct consequence of inflammation; 2. *consecutive ulcers*, or those which originate in the intestinal mucus membrane.

There are not many cases on record of primitive ulceration of this membrane. M. Scoutetten observes, that if the patient is not exhausted, and continues to live for some time, erosions of the peritoneum, at first slight, become by degrees more and more deep, and are converted into true ulcers, which may extend and destroy all the membrane, and even the subjacent tissues.* In a patient who had suffered from a venereal affection, and had experienced pains in the lumbar region, most severe at night, M. Portal found, on examination of the abdomen after death, several ulcers covered with pus in the peritoneum situated on the anterior of the lumbar vertebræ and of the kidneys. The same author cites Bouet and Paw as having seen subjects in whom the peritoneum appeared eroded to a great extent.† M. Scoutetten observed the diaphramatic portion of the peritoneum ulcerated in one case to the extent of two inches; and there is a case also mentioned by the same author, in which, after symptoms of peritonitis, the abdominal parietes were perforated at the umbilicus, and a whitish fluid of the consistence of pus escaped from the abdomen. We have before mentioned, in treating of partial peritonitis, that purulent effusions in this form of the disease occasionally escape by an opening into the stomach or intestines; from this we would infer that ulceration of this membrane may occur more frequently than is generally supposed, but there are not many cases of this description on record.

Consecutive ulceration is much more frequent, and will be fully considered in the article *Peritonitis from perforation of the serous membrane.*

2. *Morbid Appearances in Chronic Peritonitis.*—The organic changes which are the consequence of chronic peritonitis are very nearly the same as those which result from the acute form. There are some peculiarities, however, belonging to the chronic species which deserve attention. In chronic peritonitis, the redness, the result of increased vascularity, is of a darker hue, and the the larger branches of veins are more considerably dilated.‡ The peritoneum has acquired

---

* Archives Generates, tom. iv. p. 392-

† Anatomie Pathologique, tom. v. p. 126.

‡ *Armstrong*, p. 76.

a greater thickness, and the inflammation appears to have penetrated to the subjacent membranes and organs. The false membranes are very numerous and firm, and unite many coils of intestine; sometimes they form a kind of envelope which surrounds the great epiploon and the intestines, and sometimes partial pouches are formed by false membrane which contain a quantity of fluid; when these membranes are detached, we find the subjacent peritoneum less red than in acute peritonitis. In some subjects scarcely any liquid is found; in these cases the false membranes are less abundant and less thick; and the intestines are united to each other by the adhesions which the inflammation has caused them to contract, and not by intermediate albuminous layers.*

In some subjects, on the other hand, a considerable quantity of effusion is found without any false membrane on the peritoneum, which is thickened, reddish, and exhibits a multitude of dilated bloodvessels. The epiploon is red, thick, and fleshy, and sometimes contains between its laminæ transparent vesicles like hydatids, and substances resembling granulations are occasionally seen on its surface.

When the peritonitis has been of several months' duration, it sometimes occur that the abdominal parietes are not distended by effusion, but are pressed close to the intestines. The epiploon is covered with a crowd of whitish tubercles of variable size, surrounded with bloodvessels more or less developed. These tubercles may also exist on any part of the peritoneal surface: Dr. Armstrong has found them under three modifications, 1. as small miliary points semitransparent and firm; 2. as uniformly opaque bodies of a larger size, and nearly of the color and consistence of the kernel of the ripe horse-chesnut; and, lastly, as soft white substances, not unlike cut portions of the medullary matter of the brain. The first and second modifications are seated in the subserous cellular tissue, and likewise between the mucus and muscular intestinal coats; but the soft medullary variety appears to be formed in general on the free surface of the serous membrane itself.† Tubercles at first are extremely minute, but they gradually increase in size and number, and sometimes coalesce: they occasionally exceed the size of a large pea. When small, they are of considerable consistence, and adhere with such tenacity to the peritoneum, that they can only be separated by tearing this membrane; but as they increase in magnitude, they become softer, and approach in ther iappearance to pus, when they can be easily detached. After being softened, they may again become indurated, and are sometimes converted into a calcareous matter. Occasionally, when they have existed for a considerable time, the peritoneum in the intervals between the tubercles becomes of an obscure red color, or bluish or black, and presents a strong contrast with the white appearance of the tubercles. These bodies may ulcerate and give rise to perforation of the intestines; when tubercles exist, there is generally only a small quantity of fluid in the abdomen, which may be of different shades of color from a grey to a black. In most instances, where they have been found on the peritoneum, they existed simultaneously

---

******Scoutetten*, Arch. Gen. tom. 4, p. 387.**

**† Morbid Anatomy, etc.**

in other organs, especially the lungs. If the symptoms which characterize a tuberculous diathesis co-exist with signs of chronic inflammation or irritation of the peritoneum, we may have some reason to suspect a tuberculous state of this membrane.

M. Bayle has described certain bodies which he calls *granulations*, presenting a pisiform white appearance, and being of a hard consistence, not unlike miliary eruptions of the skin; but M. Broussais supposes they are nothing more than a transformation of the exuded matter which passed from a liquid to an organized state.*

Chronic irritation may produce several other morbid changes on the peritoneum or the subserous tissue, the detail of which would include nearly the whole of the morbid anatomy of this membrane. It may become cartilaginous, bony, or scirrhous. The mesenteric glands may become diseased and tuberculous. Broussais has found vesicles resembling hydatids, and extensive lardaceous depositions, in the subserous cellular tissue. This fatty matter was not only deposited under the peritoneum, but also between the laminæ of the mesentery and omentum; it was of a white or yellow appearance like tallow, and mixed with a gelatino-albuminous fluid of thinner consistence and darker color, which gave the whole mass a mottled appearance. He supposes this the result of chronic inflammation of the subserous tissue, but whether those productions are always to be considered the effects of inflammatory action is doubtful. They at all events are not the usual results of this state; and it appears necessary that a peculiar disposition to such formations must pre-exist in the constitution, which may be called into action, and determined to any particular structure or organ by the existence of chronic inflammation or irritation there.

M. Scoutetten† has described a morbid appearance which he considers as peculiar to primitive chronic peritonitis. It consists of a number of spots formed by little points, the number and close propinquity of greyish which determine the intensity of the color of the spots, which are sometimes brown or even of a black hue. These spots vary in number, and are sometimes only a line, and in other instances an inch, in extent. They are occasionally accompanied with an increased development of vessels. Minute yellowish vesicles are also sometimes seen, and an abundant serous effusion almost constantly co-exists, in general transparent, because the inflammation has not been sufficiently active to change entirely the mode of secretion.

*Treatment.*—The general principles of treatment which are applicable to other internal inflammations are equally so to this; with some modifications however, arising from the nature of the texture affected, and from its relations to the organs which it covers. We shall first consider the treatment of this disease in the infant, and afterwards in the adult, and lastly speak of the management of chronic peritonitis.

---

* History of the Chronic Phlegmasiæ of Broussais; by Hays and Griffith, vol. ii. p. 294.

† Archives Generales, tom. iv. p. 398. We have been much indebted to this author for information on the morbid anatomy of the peritoneum. This essay contains more information on this point than any author we have consulted.

1. *Treatment of Infantile Peritonitis.*—Our principal remedy in infantile peritonitis is the abstraction of blood, either general or local. In an infant under six months, though general bloodletting may often be required, yet a sufficient quantity can usually be obtained by the application of three or four leeches to the hand or foot, where we can easily control the hemorrhage, which cannot be done so effectively if the leeches be applied to the abdomen. Applied to the extremities they are nearly as efficacious in removing local inflammation in infants as when applied to the vicinity of the part affected. They seem to produce the same result as a general bloodletting, as the face and lips become pale, the pulse falters, and syncope followed by vomiting occasionally takes place. These effects are apt to be produced when general bloodletting is carried to a considerable extent; and sometimes a state of nervous agitation and general commotion is induced, which, if not speedily removed, may terminate in death. The best remedies in a case of this kind are the horizontal position, cool air, and a drop or two of the tincture of opium.*

Where general bloodletting is practised, from two to three ounces may be abstracted from an infant between six and twenty months old; at two years from three to four ounces; and when the age is above four, about five, six, or eight ounces may be drawn according to circumstances. After the inflammatory action is lowered by the abstraction of blood, advantage will be derived by establishing and keeping up an open state of the bowels, but we should avoid affecting this by irritating medicine. Small doses of calomel alone, or combined with a little of the pulv. corn. ust. cum opio, if the stomach is irritable, followed at intervals by castor-oil or emollient enemata, will in general be found to answer sufficiently well. Fomentations to the abdomen will tend much to relieve the pain, and should be often repeated and continued for some time; or we may put our little patient for a few minutes in the warm bath. If it is judged necessary to apply counter-irritation to the abdominal surface, warm flannel sprinkled with turpentine appears to us much preferable to blistering, as it produces a sufficient ruberfacient effect, without the injurious consequences which blisters often produce on infants. The recommendation of M. Billard, to remove the child from the breast, appears to us questionable. The sudden alteration in diet would be very apt to produce derangement of the stomach or bowels, a complication which would add to the danger of the patient. If the acute symptoms subside, and it seems probable from the contiuuance of abdominal tumefaction, slight dyspnœa, quick weak pulse, dry tongue, and hot skin, that the affection has passed into the chronic state, we should suspend or be sparing of evacuations; a leech or two to the abdomen may occasionally be necessary. The strength is to be supported by animal broths, arrow root, etc.; the bowels regulated by calomel in the combinations above recommended, according to circumstances. The warm bath may be occasionally useful; also counter-irritation to the abdominal surface, and in some cases mercurial inunction.

*Treatment of Peritonitis in the Adult.*—Acute peritonitis, though

---

* *Dr. Cuming*, Transactions of the King's and Queen's College of Physicians in Ireland, vol. v. p. 49.

generally attended with considerable danger, yet in the greater number of cases admits of a cure by active and early treatment. The following are the indications which we should have in view; 1. to reduce the action of the heart and vascular system; 2. to diminish the hyperæmia of the affected part; 3. to allay local and general nervous irritation; 4. to restore the secretions to a natural state, and to excite the peristaltic action of the bowels; 5. to relieve urgent symptoms. Copious and early bloodletting are the most efficient means we can employ for the reduction of vascular action. This is indeed the principal therapeutic agent in this disease, in which it can be employed to a greater extent than in inflammation of mucus surfaces. Its efficacy is greater according as it is used early, and carried to such an extent as to make a decided impression on the system. The quantity abstracted is to be regulated by our estimate of the capability of the patient's constitution to bear depletion, and not by any arbitrary rule of quantity. We have seen five or six ounces produce as decided an effect on a delicate female as thirty ounces on a robust patient. We should take the blood from a large orifice, and allow the stream to flow either until the pain is relieved, or weakness of the pulse, paleness, and tendency to syncope is induced; the apparent debility which the patient exhibits in the onset of the disease is not to deter us from active depletion. The pulse commonly rises after venesection, and becomes fuller and softer, and the patient feels relieved and lightened, rather than exhausted, by its employment. The advice which Dr. Abercrombie gives on the employment of bloodletting in this disease we have experienced the value of in many cases: viz. "to follow up the first bleeding by small bleedings at short intervals, when the effect of the first begins to subside; in this manner we prolong, as it were, the impression which is made by the first bleeding, and a twofold advantage arises from the practice—namely, that the disease is checked at an early period, and that the quantity of blood lost is, in the end, much smaller than probably would be required under other circumstances; if we allow the patient to lie after the first bleedings ten or twelve hours, or even a shorter period, the effect of it is entirely lost, and a repetition of it, to the extent of twenty ounces, may be required for producing that effect upon the disease, which by a former method might be produced by five. And, besides, the disease has been in the interval gaining ground, its duration is protracted, and the result consequently rendered more uncertain. The inflammation of a vital organ should not be lost sight of for above an hour or two at a time, until the force of it be decidedly broken; and unless this takes place within twenty-four hours, the termination must be considered as doubtful."

The efficacy of bloodletting will depend in a great measure on its early employment, but we are not to abstain from it altogether at even an advanced period of the disease. If we have not been so fortunate as to see our patient in the commencement of the attack, still if the stage of collapse has not been formed, if there is still some pulse and heat of surface, with abdominal pain and tenderness, we may abstract a moderate quantity of blood from the system with a chance of producing a good effect; or in debilitated patients apply leeches; but when the symptoms indicative of sinking are present, it would be obviously improper to take blood either generally or locally, as it would only hasten the fatal termination, and bring undeserved reproach on a valuable remedy.

Having reduced the general vascular action by the lancet, our next object should be to diminish the quantity of blood in the affected part by local bleeding, which will also assist in keeping up the constitutional effect produced by venesection. Gooch* has well observed, "that as long as the pulse is quick, full, and hard, it is in vain to take blood from the affected part; if we could completely empty its gorged capillary vessels, they would be instantly gorged again, whilst the heart and large arteries are injecting them with so much violence. On the other hand, after having reduced the force of the general circulation, the capillary vessels of the part often remain preternaturally injected. This I conclude from the fact that the patient is often not relieved till local bloodletting has been used, and then is relieved immediately."

Having allowed the patient to recover from the faintness produced by the general bleeding, the abdomen should be slightly fomented with warm water, wiped frequently dry, and leeches should be applied in numbers proportioned to the urgency of the symptoms and strength of the patient. They should be especially concentrated over the parts where most pain and tenderness on pressure exists, and after they have fallen off, fomentations with cloths dipped in warm water should be assiduously applied and repeated for some time, which will both encourage the bleeding and soothe the irritation of the inflamed parts. The application of leeches may be repeated several times, as long as any considerable soreness remains.

Either before or during the application of the leeches, and as soon as possible after the vascular action has been reduced by venesection, from five to ten grains of calomel combined with one or two of opium should be administered, which may be repeated in diminished doses every three or fours hours. By this combination, the constitutional and local irritation consequent on the inflammation, and which has a tendency to aggravate it, will be allayed by the narcotic, and the secretions, which have been more or less suspended or deranged, will be restored by the mercury, which modifies its action, and determines it to the skin, and is also supposed to equalize the circulation. After the second or third dose of this medicine, the bowels may be opened by mild aperients, aided by enemata; castor-oil, in doses of from half an ounce to an ounce in some aromatic water, may be given it the stomach is not irritable. If vomiting is urgent, the Rochelle salts with the supercarbonate of soda, in a state of effervescence with lemon juice, may be used in repeated doses, so as to produce a moderately laxative effect. Strong purgatives are highly injurious, and even a small dose of castoroil may exasperate the disease, if used previously to depletion. Having evacuated the bowels, the use of the calomel and opium should be resumed, and continued till the mercury has affected the system. As soon as sailvation is established, we have generally found the symptoms become much mitigated, and our experience accords with that of Dr. Gooch, who remarks that whenever the gums were affected in this disease, the patients invariably recovered. The establishment of mercurial action not only assists in subduing the inflammation, but may

---

* Diseases of Females, p. 45.

prevent or remove those effusions of lymph which afterwards form adhesions that are often the source of future mischief.

In addition to these means, the warm bath may occasionally be useful, or repeated fomentations to the abdomen will tend much to relieve the pain and soreness. Counter-irritation, by means of blisters, after local and general bleeding, is generally recommended; but we have not been much in the habit of employing them. While much inflammatory excitement prevails, they would prove injurious, and at any period of the disease their application would deprive us of the most important means we possess of ascertaining the degree of tenderness by pressure. The application of warm flannel dipped in turpentine we conceive a good substitute, and it will generally produce a rubefacient effect.

Oil of turpentine, which was first recommended by Dr. Brennan of Dublin in puerperal peritonitis, may be useful internally in certain forms of the disease, but we conceive it requires to be used with caution; it acts as a powerful cathartic, and at the same time excites the general system; hence it would obviously prove injurious while much heat of skin, frequent pulse, and indications of active local inflammation were present; but in a debilitated patient, after the acute symptoms have been subdued, or in cases of puerperal peritonitis accompanied with typhoid symptoms, or where general and local bleeding cannot, from the delicacy of the patient's constitution, be carried to the requisite extent, it may be employed with advantage.

During the whole course of our treatment, the strictest antiphlogistic regimen should be observed. Light farinaceous diet, in small quantities, and rice or barley-water for drink, are most suitable for the patient; but if symptoms indicative of a sinking of the vital powers should appear, wine and other tonics may be necessary. Dr. Abercrombie used injection of beef-tea and cinchona with advantage.

When we have carried venesection to a considerable extent, and have reduced the vascular action to such a degree as renders the abstraction of more blood inadmissible, if we still find pain and tenderness present, the exhibition of a full opiate, followed by fomentations or a warm poultice to the abdomen, will sometimes remove the symptoms. If vomiting is urgent, it may sometimes be checked by saline draughts with tincture of opium, or by leeching and blistering the epigastrium. When the pulse continues very frequent after the inflammation appears to be subdued, Dr. Abercrombie recommends the use of digitalis.

A tympanitic state of the abdomen at an advanced period of the disease may occur from mere loss of tone, after the inflammation has been subdued. Small quantities of wine or brandy may be given at short intervals. Frictions of the abdomen, and injections of beef-tea, bark, or sulphate of quinine, turpentine, or tincture of assafœtida, with a moderate quantity of laudanum, may be repeated every two or three hours. The bowels may be moved with mild laxatives, such as aloetic wine, or aloes and hyoscyamus, but laxatives require to be given with the utmost caution. The authorities for the tobacco injection in inflammation of the bowels are numerous; among others, De Haen, Fowler, Abercrombie, and Howship, have recommended this remedy; the latter author relates three cases in which, having tried bleeding, the warm bath, and stimulating injections without effect, the fume of tobacco cautiously injected caused a general commotion and rumbling noise in the bowels, which was soon followed by copious evacuations of fæcal matter. The patients were all saved.*

During convalescence the greatest care is necessary in order to prevent a relapse. The patient should observe the strictest temperance in his diet, and return with great caution to the use of animal food or wine. The bowels should be kept regular, the feet warm, and flannel worn next to the skin for a long time after every symptom had disappeared.

*Treatment of Chronic Peritonitis.*—Chronic inflammation of the peritoneum, when far advanced, is in most cases incurable, especially when the false membranes, and other morbid productions are considerable in quantity, or when it co-exists with a tuberculated state of the peritoneum or subjacent cellular texture, such substances being for the most part incapable of absorption; but where the effusion consists of serum, with but little or no solid productions, our chance of success is greater. Much will depend on arresting the disease at an early stage, at which time a degree of subacute inflammation often exists which will require, with some modifications, the same treatment as the acute form. At every period in this disease, when the abdominal pain and tenderness are present, and the constitution of the patient is not very much debilitated, blood may be abstracted from the system to the extent of six or eight ounces at a time, which may be repeated twice a week until those symptoms have disappeared. The application of leeches also may be frequently adopted; this will be found the most effectual mode of relieving the abdominal soreness. All fæcal accumulations should be prevented, and the bowels kept regular by the gentlest aperients or enemata, but active purgatives should be avoided as they may be productive of serious evil. The warm bath or fomentations to the abdomen may be frequently employed, and will assist much in allaying irritation and pain, and in determining to the surface. Blisters may also occasionally be applied to the abdomen, or the external application of turpentine, as recommended in the acute form, will be found useful in relieving the abdominal tenderness.

The antiphlogistic regimen is to be observed to a certian extent, and light nutritious diet, composed principally of the farinaceæ and milk, in limited quantities at a time, appears the most suitable. Muscular exertion, or pressure on the abdomen, will be found to aggravate the symptoms, and are of course to be avoided; but in some cases gentle exercise in a carriage will promote the general health. A sea voyage has been recommended, and may be useful.

During the whole course of the disease we are to guard against the supervention of acute inflammation, which may be induced by a very slight exciting cause, and is especially to be dreaded, as the patient, from his debilitated state, could not bear the evacuation which would be necessary for its removal. When the pain and soreness are mitigated, if serous effusion exists, its absorption will be promoted by diuretics. Digitalis, either in the form of infusion or tincture, may be given conjoined with the alkalis and the spirit of nitric ether; but care should be taken to excite as little irritation of the stomach or intestines as possible. We have found the induretted ointment of Lugol, mixed with equal parts of mercurial ointment, applied by friction to the abdomen

*Practical Observations, p. 19.

a powerful means of exciting absorption in cases of ascites consequent on peritonitis. In some instances it surpassed our expectation in producing the complete removal of considerable ascites in a few weeks. We have also in some cases exhibited at the same time internally the aqua mineralis iodinæ of the same author, and found it a valuable auxiliary. Broussais strongly recommends the introduction of diuretic medicines, such as the tincture of cartharides or squill, by means of friction on the skin, and it may be proper to try this mode of exciting diuresis, when, from irritability of the stomach, we cannot give diuretics internally. Anodynes may occasionally be necessary, and we should select those which do not produce constipation, such as hyoscyamus or conium. Rigid abstinence has been recommended as a means of producing absorption,* and in some cases perhaps it may be tried, but it will be improper where the patient is much debilitated by a protracted disease. Where all inflammatory symptoms have subsided, and a state of exhaustion remains while the effusion continues, tonics combined with diuretics may be cautiously tried. We have in such cases derived considerable advantage from the exhibition of the ferrum tartarization in solution, combined with the spiritus junip. comp.: it appeared to improve the patient's general health, and excite the action of the kidneys at this period of the disease. The antiphlogistic regimen will require to be relaxed a little, and more nutritious diet in small quantities may be allowed."

In the volume from which we have just quoted, is an article on "PLETHORA" a semi-pathological state, of which much was said by the older writers, though no reference is made to it, in many modern books. Dr. Barlow, of Bath the author of this dissertation, sets out with the following exordium:

"Pathology regards living actions, which depend on organized structure, and this both derives its nutrient elements from the blood, and returns to it the effete matter which the nutrient deposits displace. This connexion, subsisting without intermission so long as life endures, is so intimate, that whatever affects the condition of the blood must immediately concern the well-being of the whole frame; whence every approach to a correct pathology of the circulation must contribute to the establishment of sound principles, and improve medical science. But pathology embraces more than the mere lesions of the blood or of its circulatory apparatus; there is a nervous system as well as a vascular, which cannot be overlooked. We wish it, therefore, to be understood that in the following doctrines of plethora we have not the slightest intention of establishing an exclusive pathology, or of claiming for the facts and reasonings adduced a higher importance than they intrinsically merit.

The term *plethora* but ill expresses the state of constitution which it is used to designate, yet it would be difficult to substitute one more correct and appropriate. Indeed, no concise term could convey the

---

* Med. Chirurg. Rev. Sept. 1820, p. 187.

compound idea which requires to be represented, and which involves the conception not so much of the quantity of the circulating fluid, as of the relative proportions of its constituent parts. The term hyperæmia, as being of similar import, would not be preferable even if it were not otherwise appropriated. It is applied to denote local accumulations of blood arising from congestion or determination, in which the phenomenon results solely from the increased quantity of blood, without reference to its quality, and is, consequently, expressed with correctness by a term representing such excess.

Plethora, though inducing a morbid condition of the body, has not in general been designated a disease, nor treated of by practical writers, save in connexion with some special malady attendant on or derived from it. Linnæus has given it a place in his nosology in the class *Deformes* and order *Decolores*, and thus defines it: "Rubedo corporis a distensis vasis sanguineis, cum dyspnœa." Sagar also admits it in the class *Cachexiæ*, and order *Intumescentiæ*, giving not only a definition but a description also: "Intumescentia universalis, proportionata et æquabilis ex abundantia sanguinis." "Amystidis et ventris cultus, pulsus plenus aut suppressus, venarum amplitudo conspicua, æstus et gravitas totius corporis, respirandi difficultas, lassitudo spontanea, stupor, artuum, somni turbati, partes epidelio tectæ ruberrimæ, temperamentum sublaxum, sanguineum." The former of these would seem to denote rather special disease of the lungs, and the latter to characterize obesity. Neither is calculated to convey a clear or correct notion of the morbid condition which excess of blood occasions. As this condition is present in many diseases, and actually gives rise to several; and as, even in cases where it does not exist, it is of importance to be assured of the negative, it cannot but be deemed an important part of pathological science to trace the circumstances which generate a state of plethora, and the phenomena which indicate it, so as to be prepared both to recognize it when present, and to apply means suitable and adequate for its correction."

We concur entirely in these views. They present the animal body in its true compound character, of solids and fluids, perpetually acting and reacting on each other, both in health and disease; and bring us to the *apriori* conclusion, that every problem in physiology and pathology is essentially complicated, and requires many *data* for its solution. We, also, concur in the views of the following paragraph, which cannot be too strongly impressed on the minds of medical practitioners, who by a timely *obtrusion* of advice upon those, whose health they superintend, may often avert the full establishment of a disease, which they would be unable at a more advanced stage to subdue.

"It is easy to understand why the investigation of disease has for the most part commenced with that period when a nosological malady is considered as formed. Until this period arrives medical aid is rarely sought for, the patient's consciousness of indisposition being aroused only when the functions of health become so impeded as to fail sensibly in their accustomed exercise. The accession is then referred in general to some exciting cause, real or imaginary, and is considered as having commenced at the time when the imputed cause was supposed to operate, a state of previous good health being assumed. Thus suddenness of accession has been regarded as a character of most acute diseases, and the period when the accession has been first observed has been regarded as identical with the commencement of disease. Yet it may be doubted whether any disease, excepting such as results from a morbific poison, ever takes place suddenly or without previous derangement of general health, cognizable by its appropriate manifestations, and capable of being corrected so as to obviate the morbid accessions to which it leads. If this can be demonstrated, it is clear that this introductory stage of disease is of the highest importance, as being that to which prophylactic treatment can be most benefically directed, and also as forming a part of the ensuing disease essential to its complete history, and without a knowledge of which its intimate nature or the series of morbid changes never can be thoroughly understood."

Our author divides plethora into *absolute and relative.* "The former implying that the redundancy exceeds what the healthy state of the individuals constitution would require or bear; the latter, that without being absolutely excessive, it is relatively so, in reference to the deficient powers of the constitution for appropriating or otherwise disposing of it." On the progress of absolute plethora he has the following observations:

"We have stated that absolute plethora is the parent of pure inflammation. If there be no plethora, inflammation will not be excited by slight causes; or if it be aroused through the operation of an exciting cause, it will be mild and easily subdued. The severity of inflammation, too, will, *cæteris paribus*, be proportionate to the degree of plethora pre-existing. Previously to the occurrence of febrile or inflammatory action, there is always a sensible interval of disease marked by evidences of diminished power in the arterial system, the oppressed and irregular actions of which evince its inadequacy to carry on the circulation with its wonted vigor. The pulse, if examined, will be found low, oppressed, irregular; which state passes progressively into one of permanently increased action of fever. Multiplied observations have satisfied us both that the stage of disease here mentioned precedes that of febrile action, and that the morbid actions indicated by the pulse succeed each other in the order here announced; the first being that of feebleness or overloaded power, the second of irregularity, and the third of permanently quickened circulation. It has been already stated how incipient plethora, when the redundancy is no longer disposed of by the healthy action of the several organs of appropriation, and when these can no longer perform steadily the increased labor, gives rise to the state of feebleness now under discussion. To comprehend the nature of these several changes is not difficult, it being readily explicable by reference to well-known laws of vital action.

When redundancy of nutritive matter first occurs, its immediate effect is to promote more vigorous circulation, and to excite to increased actions the several capillaries, especially those engaged in nutrient secretion. The peculiar stimulus of the nutritive matter excites these actions; their end is to dispose of the redundancy by natural appropriation, and the effect on the frame in the first instance is only that of increased volume and exuberant health. But to all vital actions, and the degree to which they can be continuously sustained, there is a limit; and when increased beyond this they after a time become relaxed, sinking even below the natural standard. By incipient plethora increased actions are excited which at first differ from perfectly healthy actions only in degree. In time, however, and especially if the plethoric state be kept up by excessive nutriment, they become enfeebled and abate; then it is that the pulse, which antecedently was fuller and stronger than natural, first becomes low and oppressed. The disposal of redundancy by increased action of healthful processes proving inadequate, from inability of the vital powers, to continue it, other efforts are now needed; and as these, though in their tendency corrective of what is amiss, no longer resemble the healthy actions, they must be considered as morbid; disease being the result of their institution. While we thus regard them, however, we should never lose sight of the corrective tendency which originally belongs to them, nor fail to profit by the curative indications which the efforts of nature point out. The minute changes so induced we pretend not wholly to explain, though many of them are readily intelligible; but the cognizable phenomena are sufficiently obvious to mark their connexion and dependency, and thus to establish a rational theory of the course which nature pursues.

Relief by increased nutrient secretion not sufficing, a more general excitement seems now required, the object of which may be to call into more vigorous exercise the several excretories; and a state of generally increased or febrile action ensues. The state of irregularity is obviously the transition from the state of feebleness to that of permanently increased action or fever; and the end of the latter is to get rid of the original cause of disturbance. It is in proof of the correctness of these views that if in the stage of feebleness depletion and abstinence be restored to, the feebleness disappears, natural vigor is renewed, and health is restored, without any febrile action being instituted; while if this state be treated with stimulants and nutritive diet, febrile or inflammatory action is sure to result. We wish here to observe that in these remarks we use the term *fever* to denote, not any of the specific diseases known by that name, but simple pyrexia, characterised by a quick pulse, hot skin, and furred tongue, being the constitutional state attendant on the ordinary phlegmasiæ, and so generally, though a radical misconception, denominated symptomatic fever. As the stage of feebleness is relievable by evacuations and abstinence, so are those of irregular action and of febrile excitement to be remedied by the same means; and if these be duly employed, any or all of these morbid conditions may be promptly corrected withont specific disease or local lesion of any kind ensuing. But if they be not employed, and more especially if, through misconception, stimulants be used and nutritive diet continued, then febrile action becomes more determinately aroused, and some specific disease of an inflammatory character is engendered ; or else, if the constitution be, from natural inertness or the extent of labor to be performed, unequal to the effort necessary for generating a state of fever in inflammation, the general health progressively declines, the powers of life become enfeebled, and the constitution finally sinks under some of the complicated forms of chronic disease. When under the former of these results active fever or inflammation occurs, it is in general speedily subjected to medical treatment; and as opinions vary but little respecting the measures to be pursued under such circumstances, while the urgency of disease requires them to be employed with proportionate vigor, it seldom happens that this stage of disease is improperly treated, at least so far as regards the use of evacuants. At this time the propriety and necessity of active treatment and of depletory practice are admitted on all sides; yet previously to the acute attack a deviation from a healthy state existed, which admitted of detection, and which as clearly indicated the propriety of some depletion, though it might not demand it so imperatively, nor require it to the same extent, as when the acute attack had supervened. Were this introductory stage relieved by adequate depletion and other suitable means, there can be little doubt that the accession of acute disease might in every instance be averted, or at least so greatly mitigated as to be free from all danger.

It appears from all that has been stated, that incipient plethora in a healthy constitution excites at first only increased energies of healthy functions, manifested in the increased bulk and more florid aspect which such persons usually present, and in the evidences of more vigorous circulation which the pulse affords; that this state of increased healthy action, if urged too far, declines into one of diminished power, still evinced by the pulse, which then becomes low, oppressed, and irregular; and that, if these progressive changes be overlooked or unrelieved, a state of permanent excitement succeeds, such as constitutes fever or inflammation. It is obvious, then, that the increase of bulk and more florid aspect in which so many exult as evincing sound health, and which they endeavor by all the aids of good living to promote, is not a source of unmixed congratulation; but that, on the contrary, it deserves to be regarded with no slight suspicion, as actually verging on consequences by which both health and life may be forfeited. Up to this period, however, disease cannot be said to have commenced, however it may be approached; and reduction of diet, with free bowels and increase of active exercise, would suffice for getting back to sounder health without any need of medical interference. When abatement of heathful energies becomes evinced by a low and oppressed pulse, diseased acactions may be said to commence, and when the stage of irregular action ensues, sensible progress may be considered as made towards the establishment of febrile action and inflammatory disease."

Relative plethora is more difficult to characterise. It is often formed in a very insidious manner.

"Health is observed to be less perfect; there is occasional languor and disinclination to the customary exertions, with irregular distribution of blood as marked by coldness of feet and variable countenance; the bowels are irregular, the appetite is capricious or fails, and both flesh and strength decline.

At the earlier periods of this state the pulse will be found weak, often irregular. Sooner or latter a febrile or inflammatory state ensues marked by a quickened circulation, some increase of temperature, and a white or furred tongue. This state may continue for an indefinite period, and be subject to frequent flucutuations; most frequently in course of time some part more particularly suffers, a local ailment arises which excites attention, and to which, when discovered, the constitutional indisposition is most commonly ascribed. In order to judge correctly of this condition of plethora, it is is necessary to mark particularly the accordance of its phenomena with those which absolute plethora presents, especially in the changes which the pulse undergoes. This, here also, is at first feeble and oppressed, then irregular, and finally it becomes permanently quickened.

As the incipient lowness of the pulse is the symptom which so generally misleads, conveying the impression of debility, and suggesting the employment of tonic and stimulant remedies, it is highly necessary to distinguish it from a pulse of pure debility. Happily there are other circumstances besides the pulse to direct the judgment in this respect; other obvious derangements co-exist, displaying a harmony of symptoms, which, taken collectively, establish beyond a doubt the existing condition of the vascular system. But the language which they speak is not always understood; their warning voice is unheeded; and the deceptive lowness of pulse is suffered to counteract the evidences which the other coexisting symptoms display. On this account it is that we dwell so much on an accurate discrimination of the indications which the pulse affords; not so much for the positive evidences which it furnishes—for there are others much less equivocal, and far more worthy of being relied on—as that, when this peculiar lowness exists, it shall not be suffered to bias the judgment or to divert the practitioner from those measures by which alone the morbid condition referred to can be corrected. A little accuracy in the mode of examination, with attention to the impressions made on the finger, will readily detect the peculiarities which we have stated; and when familiarised to the touch by habitual perception, there can be little difficulty in distinguishing them. In the condition of which we are treating, if firm pressure be made on the artery, it will be found to resist beyond what its apparent feebleness would indicate, and, on gradually withdrawing the finger, to rebound with a force much greater than would at first be imagined. If the pressure and relaxation be a few times alternated, the sensation will be rendered more distinct. This inherent firmness is sufficiently indicative of the condition existing, and affords the best assurance of depletion being well borne. As we before stated, however, and as will be hereafter more distinctly pointed out, there are other evidences to confirm what the pulse proclaims.

The state of irregularity of the pulse also requires precision in determining both its character and extent. It may be perceived as affecting both the force and frequency of the pulsations; and the irregularity of force, or that in which the artery makes a few strong pulsations, as if by a transient effort, and again relapses into a state of oppressed and diminished action, indicates, so far as we have been able to discover, a nearer approach to febrile or inflammatory excitement than the irregularity of frequency only; repeated observation of which fact has led us to infer that this stage of irregularity is the connecting link between the stages of feebleness and of permanently increased action, and that it consists of the early but yet imperfect efforts of the vascular system to form this latter stage; for when the febrile or inflammatory action is fully formed, the irregularity is no longer perceived."

It is oftener with relative than absolute plethora, that we find the union of those elements which go to vitiate the blood. These arise from defective secretion and excretion, which may, in fact, often be the *cause* of the plethora, and must always modify its phenomena, by depressing and otherwise disturbing the powers of life. This condition is frequently the lot of the sedentary;—for muscular exercise is the great exciter of the excretions. This state of sanguinous contamination

"Is characterised by great sallowness of aspect and duskiness of skin; the pulse is low and compressible; the surface of the body is in general harsh, dry, and deficient in natural transpiration; the tongue is for the most part moist and clean; the appetite capricious, often voracious; the alvine evacuations are inveterately foul, exhibiting no traces of healthy secretion; the urine is high colored, depositing a dark sediment, and often very fetid; even the perspiration has frequently an offensive odor, and gives a dusky tinge to the linen which absorbs it."

The treatment of absolute plethora, must be by moderate bloodlettings frequently repeated, a reduced and vegetable diet, occasional purgatives of the chologogue and hydrogogue kind, active even laborious exercise, and reduction in the hours of sleep. Of the whole, the two laest are, perhaps, the most effective. Bloodletting alone will not answer. It seems, indeed, to give a new impulse to the function of sanguification. Such is the law of the animal body, otherwise its waste, under various casualties would not be supplied in due season. In many instances ,bleeding is not required, because the physician perceives the *rising* plethora, and by advising other means than bloodletting, arrests the hyperœmia. If not consulted in time, he will find the lancet indispensable; but he should never draw blood in these cases, to syncope. Small, repeated bleedings, will be far more efficient. The vessels will accommodate themselves to such reductions, and not, to speak figuratively, invoke the aid of the *vis conservatrix*, for a new supply.

Our author dwells at considerable length on the principles and modes of treatment of relative plethora, which is exceedingly apt, if neglected, to eventuate in fever and inflammation. We trust to be excused, for presenting our readers with the parallel he has drawn, between the therapeutic indications in the two varieties of the state under consideration.

"It has been shown that the derangement of circulation incident to a state of plethora, whether absolute or relative, manifest an uniformity which serves to mark the nature of the morbid changes induced, and to illustrate the processes which nature institutes in her endeavors to effect her own relief. Redundancy of nutritive matter first excites the healthy functions to increased energy, tending to its appropription by natural secretion; when too much urged this energy abates, and feebleness of arterial action ensues; to this succeeds a state of general excitement, such as is expressed by the term fever; and finally, some local congestion or inflammation occurs, producing what is called a specific disease. From this view the dependency both of the general fever and local affection on the constitutional state is at once perceived; and the treatment by wh ch these are severally relieved acquires thence a clearness and consistency which no speculative theory of disease has ever yet imparted. For absolute plethora in its simple state, it has been shown that depletion and abstinence, the remedies directly suggested by a knowledge of its cause, are those which experience proves to be most efficient, and which may alone give full relief. For the active fever and local inflammation induced by this condition, the same means are essential, and are still the most powerfnl of all that can be employed; though when these derivative maladies occur, other evacuations besides that of blood are needed, it being here required to call forth the energies of ali excretories of the system in aid of those constitutional efforts by which the febrile excitement is aroused. In relative plethora the morbid condition of the system is less simple, yet it still corresponds with the other throughout. Owing to the duration of morbid actions antecedently to the occurrence of febrile excitement, the constitutional derangements are more extensive; more functions are depraved; the difficulty of restoring these severally to a healthy state is increased; and a longer time is required to correct their aberrations and renovate their powers. During this period, too, various disturbances, originating in depravation of nervous function, become intermixed with the ordinary symptoms, complicating and obscuring the whole. To these latter we shall have occasion to advert more particularly by-and-bye. In absolute plethora the purpose is simple. If the redundancy of nutriment be diminished, and a fresh supply withheld—in other words, if blood-letting and abstinence be carried to the requisite extent, the powers of nature, thus relieved, are amply sufficient to re-adjust all that is amiss, and perfect health becomes restored.

No arteficial stimulus is here needed to arouse the natural powers; but, on the contrary, the chief care is to restrain them within salutary bounds. In relative plethora, while depletion and adaptation of diet are equally necessary as in positive, more care is necessary for maintaining in due activity the several secretories, and some degree of stimulant treatment requires to be combined, in order to excite and support those increased energies of the system by which alone this condition can ever be thoroughly rectified. Ordinary stimulants, however, which merely excite the heart and larger vessels, are inadequate to this end. Nay, they do mischief; for they lead to no effectual exercise of power, and from that which they do excite, exhaustion rather than benefit results. The great deficiency of power which here prevails is in the capillary vessels, and to depravation, abatement, or suspension of their functions, may all be the coincident derangements of the frame be readily traced. The two most essential processes of animal life are nutrition and excretion. These are exclusively performed by capillary vessels, on the due energy of which they are dependent. When from any debilitating cause these minute vessels fail in power, or become obstructed, both nutrition and excretion must become impeded; and hence we can understand why emaciation often takes place, even where there is a redundancy of nutritive matter in the general circulation, available for nutrition if the capillaries could so dispose of it. It is hence also intelligible, how a debilitating impression made on the system has, through abatement or suspension of the capillary function, the direct effect of throwing back on the mass of blood that which in ordinary course the capillaries would have disposed of, and of thus inducing a state of relative plethora. It is clear from this view, that the object of treatment in relative plethora is not merely to remove the relative excess, although this demands its full share of attention; but also to renew the activity of the capillaries in order, first to re-estabish their healthy functions as essential to the well-being of the general constitution; and secondly, by thus providing for the just appropriation of the nutritive matter supplied by the blood, and supply without which health cannot subsist, and which the occurrence of plethora, however induced, never fails to disturb. We have stated that ordinary stimulants, which produce but transient excitement of the heart and larger vessels, are not the remedies here needed. What we require is an agency which without exciting inordinately the general circulation, is capable of acting on the capillary vessels, and of arousing them to a renewal of their several functions both secretory and excretory. Such a remedy we happily passes in mercury, which is signally endued with this peculiar property, and which, in consequence, affords a powerful agency in relieving the larger bloodvessels, when overcharged, from part of their excessive load, by causing it to be diffused more freely through their numerous and wide-spreading ramifications; and when the aggregate capacity of the minute vessels thus expanded and restored to the exercise of their natural functions, is estimated, there will be little difficulty in comprehending how mercury proves so powerful an auxiliary of bloodletting in relieving an overcharged state of the general circulation, as well as in correcting the congestions and other derangements to which this state gives rise. If the effects of mercury on the animal frame be examined, it will be seen that, however diversified, they all correspond in one respect—namely, in evincing increased action of capillary vessels. Mercury has the effect of increasing almost every secretion, and by capillary vessels all secretion is performed. It promotes transpiration by the skin, diuresis, and secretion of bile; it more especially excites the salivary glands; and there is resonable ground of conjecture that it similarly affects the pancreas, to the increased secretion of which gland mercurial diarrhœa has been with some plausibility ascribed. It increases the intestinal discharges independently of its direct action on the bowels when taken internally, for mercurial frictions are well known occasionally to purge. In every effect which mercury induces this common character is observable, that capillary action is more or less excited. Now a renewal of capillary action is what has been shown to be needful for correcting the derangements incident to relative plethora, and hence the inestimable value of this remedy when judiciously administered, in restoring to health those who suffer under such derangements.

In absolute plethora the febrile action aroused suffices to impel blood into every extreme vessel, and thus to equalize circulation by extending it to every part where nature requires its full activity; and to effect this seems to be the great end for which febrile excitement is instituted by nature. In relative plethora this effort is more feeble, the natural powers being weaker, (indeed it is in this natural weakness that relative plethora has its origin;) and as every day but adds to the labor to be performed, while under the general depravation of habit the natural powers tend to further decline, we have little difficulty in comprehending why this state does not become aroused to those energetic efforts which are so often witnessed in the active fever of absolute plethora. To excite such effects then by the peculiar stimulus of mercury becomes an essential part of the treatment of relative plethora; and when in the correction of this state mercury is used with the necessary discrimination, and in aid of bloodletring, no course of medical treatment can be more successful, nor more strikingly illustrative of the principles by which it is guided, than that which this combination of depletory and stimulant agency supplies. The principle here adduced is confirmed by all that we know of the action of mercury, whether in its abuse or its use. Consistently with the views here presented, this action ought to be salutary only when the labor to be performed is not beyond what the animal powers are capable of sustaining. If the labor be too great, if inordinate plethora prevail, then must the excitement of mercury fail to effect its object, and the animal powers, exhausted by the vain effort, must still further decline. This operation of mercury corresponds exactly with the course of active fever where no bloodletting is employed. The powers exercised are in either case considerable, but the labor is disproportionate, and they sink under it. But if the labor be lightened by lowering the nutritive quality of the blood, (and this can only be effected with the necessary promptitude by blood-letting,) then will both febrile action and that of mercury accomplish all that is required from them."

We shall terminate our notice of Dr. Barlow's article with his remarks on sanguineous vitiation from defect of excretion.

"Before concluding this article, it is necessary to offer a few remarks on the vitiation of blood which arises from redundancy of excrementitious matter, regarding this in its simple state, unconnected with the various degrees of plethora with which it is so continually combined. In doing so the motive is not so much to designate the morbid condition as likely on its own account to become an express object of medical discipline, as to establish the principles which should govern its treatment, when, as continually happens, it is found combined with other derangements. The general phenomena which denote this condition are, a sallow aspect and dusky skin; the pulse low, soft, and compressible; the surface of the body for the most part harsh, dry, and obviously deficient in natural transpiration; the tongue moist, clear, red; the appetite capricious, often craving and voracious, with an endless train of dyspeptic ailments; the alvine discharges inveterately foul, dark, slimy, pitch-like, and exhibiting no trace of healthy fæces; the urine high-colored, depositing more or less of dark, often fetid sediment; these, with decline of flesh and strength, are the general characteristics of this state. The condition itself we believe to arise from the accumulation in the blood of excrementitious matter imperfectly discharged, and the depraved state of the several secretions we regard as resulting from the labored though ineffectual effort of the constitution to accomplish through their agency, its own purification. It may give a clearer conception of this condition to contrast its phenomena with those which characterize nutritive plethora. In the latter the general aspect is more full and florrid; the surface is hot and dry, or inclining to moisture; the pulse hard and frequent, or full strong, and bounding; the tongue white and furred; the stomach inclining to nausea, with thirst; the stools feculent, though foul, and charged with mucus.

In specifying the symptoms of excrementitious accumulations, it may be imagined that we have included several which belong to the different forms of hepatic diserse. That they are frequently regarded as evidences of diseased liver, and treated accordingly, we are well aware; but that they are certainly to be met with where there is no organic lesion of this viscera, nor any particular functional disturbance of it, we are fully convinced. It is true that the functions of the liver are, in common with those of the whole alimentary canal, greatly depraved, but they are so not from any primary defect or derangement of their own powers, but from being required to act inordinately to a vitiated mass which is seduous to purify.

According as nutritive plethora becomes more or less combined this state, the constitutional efforts increase, and various degrees of febrile and inflammatory excitement ensue. In proportion as this excitement is energetic, and as measures of suitable activity are employed for its relief, the vitiated state of the blood becomes corrected, the secretions and excretions improve, and the general health and strength amend. The increased secretions form the bowels seem to be the discharge by which nature aims of getting rid of such impurities. To promote them, therefore, by suitable purgatives, at the same time supporting the strength by moderately nutritive diet, is the first indication.

When relief, to a certain extent, is thus afforded, the powers of the constitution rally, and a febrile effort is made to assist in the work of purification. According as this advances, depletion should be more active and the diet less stimulating. When sufficient excitement exists to warrant the employment of blood-letting, we may then consider the curative process as in the most favorable train. Perhaps the powers of the constitution are hardly adequate to rectify any high degree of this derangement without the extraordinary effects which a state of febrile excitement supplies, and hence we see experienced practitioners often hail the appearance of febrile symptoms in chronic maladies as announcing a more remediable form of disease.

In every view that can be taken of this condition, it must be considered as intimately connected with the state of cuticular excretion, from defect of which it is more likely to arise than from any other cause. When we reflect on the large amount discharged by this excretory under a healthy state of the system, and that, according to accurate experiments, more than one half the ingesta is carried off by transpiration, it will be readily conceived that great excrementitious accumulation must result from impeded cuticular excretion. This matter being in consequence thrown in inordinate quantity on the other excretories, it can excite little surprise that their ordinary functions should be thence disturbed, or that their several secretions should present a morbid character.

A constitution naturally feeble, especially if exercise be inadequately taken, sends the blood to the surface too languidly for the exhalent arteries to act with full power, whence excrementitious accumulation commences; the effect of this being directly debilitating, it serves to aggravate the cause, and thus the foundation is laid for a broken constitution and many inveterate chronic diseases. The best preventive of this diseased condition is, unquestionably, exercise, and in slighter degrees of it this would also be the most effectual cure; but when great depravation of habit has already taken place, there is neither the capability of taking adequate exercise, nor would it alone, under such circumstances, succeed. To be effectual it should be carried to the extent of producing some moisture on the skin, as the only sure evidence of the blood being impelled with sufficient force into the capillary vessels. The various forms of warm-bathing are of great value, especially those which combine frictions and other means of softening and detaching hardened cuticle. That of the Russian vapor-bath, noticed by northern travelers, and so accurately described by the late Dr. Clarke, would appear eminently calculated for establishing a healthy state of skin, and an adequate activity of cuticular excretion."

Dr. Venables of London, is the author of the article organic diseases of the liver. We shall extract his experience and observations, on the iodurets of mercury, in some of these and several other organic diseases, as valuable to our readers. On a former occasion we made known the effects of iodine in enlargements of the spleen, according to the trials of one of the most able physicians of the south west, Dr. Cartright; and in various numbers of the Journal, we have inserted notices of the influence of the same substance, in different structural affections. The compound of that medicine with mercury, it would seem from the experience of Dr. V., is much more effective than its solitary administration. But let him speak for himself.

"Many years since it occurred to the writer that the powers of mercury might be greatly enhanced by combination with iodine, in accordance with an acknowledged rule or principle in pharmacology, that the virtues of remedies exciting singly a similar specific agency, would be greatly increased by combination; and the preparation of the proto- and deueto-iodurets of mercury* were consequently administered of various tumors and enlargements; such as bronchocele, hepatic, and other visceral enlargements, and indeed all descriptions of abdominal swellings. The results were highly satisfactory. One or two cases even of white swelling and hip-joint disease perfectly recovered by the use of the iodurets of mercury with the means to be presently detailed. It is, however, in glandular swellings and in the vascular congestions of parenchymatous structures, and the consecutive organic changes which these structures undergo, that this combination is most effectual. In a treatise on organic disease, printed in 1824, we have entered fully upon this subject and the different objects to be kept in view.

More recently we have had some opportunities of again proving the efficacy of this preparation. A young woman in the country began to swell about the abdomen, and was considered pregnant by her medical attendant. She came to town, and a medical friend was requested

---

* The proto- and deuto-iodurets of mercury may be prepared extemporaneously, by mixing calomel or oxymuriate of mercury with the equivalent of hydriodate of potass; an interchange of principles takes place even in the dry way. It is of course intermixed with muriate of potass; which might be washed out if it were of importance to free the preparation from such an impurity.

to attend her in her accouchment. He, however, doubted the pregnancy, and her time having elapsed without any diminution of size, calomel and hydriodate of potass were given, and an abdominal swelling of nearly eleven months' duration, and which confined the patient to bed for more than two months, was reduced in about three weeks and the patient restored to perfect health. A second instance occurred in the practice of another medical friend. It was an abdominal swelling, probably of an ovarian description. We recommended the plan pursued in the last case. It was, however, some time before it could be put into execution, and the swelling remained unabated. At last, however, it was adopted, and in about three or four weeks the abdomen was reduced to its natural size. This patient, however, died, (the constitution having been completely broken up by previous dissipation,) but on examination no indication whatever could be traced of the part which had been enlarged. One ovary, probably the diseased one, had entirely disappeared.

The iodurets affect the mouth in the same way as the more ordinary preparations of this mineral. They may be given in similar doses, and the deuto-ioduret in larger doses than the oxymuriate. They may also be introduced by friction; but the deuto-ioduret almost always produces a bright scarlet efflorescence of the skin, which mostly terminates in desquamation. The precautions as to correctives, when considering mercury generally, are equally applicable to the iodurets.

Some constitutions powerfully resist the mercurial action; such stubborn resistance is to be overcome, to plethoric habits, by bleeding, by nauseating doses of emetics, by opiates, and other well-known means. The introduction of mercury also is often attended with a febrile irritation or excitement, which, reacting on the diseased viscus, counteracts whatever benefit might otherwise be obtained. This must be kept in subjection by bleeding and other antiphlogistic measures, and by local measures which will also prove auxiliary upon other principles.

The affections which will be benefited by this plan are hyperæmia of every description, hypertrophy and all sorts of enlargement, induration, scirrhus, tubercular conditions, even the scrofulous, and most adventitious deposits, excepting the osseous, and even this perhaps if incipient. Its efficacy is questionable in abscess, ulceration, and cancer; but still it may be likewise questioned whether the iodurets should be wholly proscribed in such affections were we satisfied of their existence. It is not improbable that if the absorption of the diseased structure were effected, healthy deposition might be substituted in its stead.

It may be inquired for what length of time the mercurial preparations should be continued. Mercury, it has been already observed, not only excites a febrile irritation, but also exerts a noxious influence upon the economy at large, and this influence is directly as the quantity and its effects. Therefore, the object is to induce the specific effects with as little irritation as possible, and with the least possible quantum of the mineral. But in many instances the effects having been induced, the mercury must be laid aside, and the disease will remain stationary; and the practitioner must again and again have recourse to the remedy. Each succeeding application to mercury is attended with severer consequences, and it often happens that on the subsidence of the organic affection the patient finds it effected only by a total breaking up of the constitution, and a fatal sacrifice of health. The debilitating or rather exhausting effects of mercurial courses are too generally understood to need any comments in this place. Upon reviewing these facts it occurred to the writer that the good effects resulting from the iodurets of mercury might be kept up and a beneficial influence exerted upon the disease by some other of the metallic iodurets. With this view, upon the first indication of the mercurial influence, whether the development of the specific influence upon the gums, or a reduction of the disease, the ioduret of iron or of zinc were given, or the hydriodates of these metals in solution. The result was extremely satisfactory; and morbid conditions which had been but slightly affected by the mercurial iodurets were completely dispersed under the ioduret of iron or of zinc. These preparations will be found much better adapted to weakly, irritable, and leucophlegmatic habits. Of these two the ioduret of iron is perhaps the most irritating and inflammatory, that of zinc the least so. They may be given either in pills, or in solution, as hydriodates, readily by mixing a solution of the equivalent of sulphate of iron or of zinc with one of hydriodate of potass; an interchange of principles and the formation of the new salts, hydriodate of iron or zinc, is the result.* Whatever objections may be offered to the employment of the mercurial iodurets in cancerous, scrofulous, and other cachectic diseases of the liver, none such can be urged against those of iron and zinc, and therefore the practitioner may safely appeal to them under the assurance that he is not employing a destructive or injurious remedy. The doses of these salts must be regulated in a great measure by their effects. From one grain to ten according to circumstances, may be given three or four times a day; the practitioner recollecting that the smallest doses should be tried at first. Another circumstance to be attended to is that the system becomes blunted by habituation to a remedy. Increasing the dose is often carried to an extreme without benefit, and frequently not without injury. The susceptibility cannot be kept up by overdoses, but when dormant it may be awakened by a temporary suspension of the medicine. Thus, when the average extent of dose fails to produce its accustomed effects, the medicine should be laid aside for a time; and when a respite has been thus granted to the system, we shall find all its sensibility restored, and we may again resort to our means, confident of finding the usual susceptibility to impression.

The state of the urine has been noticed in a previous part of this article, and an examination of it will often afford useful information. There are two conditions of it, however, which deserve attention in hepatic diseases, namely, an excess of urea, and its ready coagulation by heat. This latter property arises from albumen or chyle, and is often present in the dropsies consequent upon hepatic disorder. It may be laid down generally that such conditions forbid any active use of mercury. It betokens a state of system altogether hostile to the use of this mineral. But the metallic iodurets just considered,

---

*In this case sulphate of potass remains in the mixture, but the impurity is of no moment according to the writer's experience, are not liable to the same inconveniences.

During the pursuance of all the above means it will be necessary to attend not only to all the functions, but to the condition of the organs which perform them. When we find urea in excess, or the curdy coagulation depending on the presence of chyle, there exists an irritability of system which must be soothed by morphia, hyoscyamus, and the acetic extract of colchicum, which will be found a most valuable auxiliary under such circumstances. The denser coagulation arising from albumen indicates not only irritability, but an excitability of the phlogistic character, which must be subdued by venesection (especially if attended with local pain) and other suitable modes of evacuation. It is almost unnecessary to observe that the digestive functions should be closely watched, and the condition of the organs closely attended to, and any degree of aberration immediately corrected. Hence the advantage of occasional stomachies, etc. Should the disorders of organs secondarily affected remain unheeded they will soon, by their reaction, aggravate all the symptoms of the primary disease.

These extracts from different pens, devoted to different articles, in the "Cyclopædia of Practical Medicine" will enable our readers to form an idea of the value it might be to them. As a comprehensive digest of medical science, down to the year 1835, it cannot fail to be peculiarly useful to gentlemen in remote places, where of course they cannot have access to the works which have been made contributory to this, and to all such we would recommend it with earnestness.

In conclusion, we refer to the History of Medicine, prefixed to the first volume, and extended through to nearly 800 pages. It is from the pens of Dr. Bostock, and of professor Alison of the University of Edinburg. The former brings the subject down to the close of the last century, and the latter completes it, with a dissertation on the progress of medical improvement, from that period to the present time. Such a work is wanted by the physicians of the west and south, as but few books on the history of medicine are found among us. In glancing over this introduction, we were struck with the fact, that *American* medicine is no where mentioned; and that no American physician is named, but Dr. Rush, and he a single time only, without a citation of the title of his works, or the country to which he belonged! We cannot be insensible to the humiliating truth, that we have not contributed equally with our elder brethren of Europe, to that rapid advancement of medical science, which has signalized the last half century, beyond every other period; nor can we affirm, that American physicians have manifested the variety of experimental research and patient observation, the literary cultivation, and the devotedness to improvement, which have carried forward the profession in France, Germany and Great Britian and Ireland. Still, with all their remissness, they have made many substantial contributions to the stock of ætiological and therapeutic knowledge, and it is difficult to account for the omission of any reference to them, but on a principle of national prejudice. Let us not, however, complain of injustice on debateable ground; but rather recollect what our duties are, and labor to perform them with greater diligence and ingenuity, than we have heretofore done or are now doing. The great fault of the American profession, is want of *concentrativeness*, and of a taste for experimental inquiries. But few of us have minds appropriated to a single object—we are stimulated by a thousand, which keep us in motion, but not advancing; he who turns round and round on his heel, may be industrious, but can have no progression. The number of our physicians is great, and in general they are sagacious and intrepid practitioners, but they must be imbued with a new and deeper spirit of inquiry, before they can place their profession on the elevated level it has attained in the old world.

